# The 2017–19 activity at Mount Agung in Bali (Indonesia): Intense unrest, monitoring, crisis response, evacuation, and eruption

**DOI:** 10.1038/s41598-019-45295-9

**Published:** 2019-06-20

**Authors:** D. K. Syahbana, K. Kasbani, G. Suantika, O. Prambada, A. S. Andreas, U. B. Saing, S. L. Kunrat, S. Andreastuti, M. Martanto, E. Kriswati, Y. Suparman, H. Humaida, S. Ogburn, P. J. Kelly, J. Wellik, H. M. N. Wright, J. D. Pesicek, R. Wessels, C. Kern, M. Lisowski, A. Diefenbach, M. Poland, F. Beauducel, J. Pallister, R. G. Vaughan, J. B. Lowenstern

**Affiliations:** 1 Center for Volcanology and Geological Hazards Mitigation, Geological Agency, Ministry of Energy and Mineral Resources, Bandung, Indonesia; 2 U.S. Geological Survey, Volcano Disaster Assistance Program, Vancouver, WA USA; 3 Institut de Physique du Globe de Paris (IPGP), Paris, France; 4 Institut des Sciences de la Terre (ISTerre/IRD), Grenoble, France; 5 U.S. Geological Survey, Astrogeology Science Center, Flagstaff, Arizona USA

**Keywords:** Volcanology, Natural hazards, Volcanology

## Abstract

After 53 years of quiescence, Mount Agung awoke in August 2017, with intense seismicity, measurable ground deformation, and thermal anomalies in the summit crater. Although the seismic unrest peaked in late September and early October, the volcano did not start erupting until 21 November. The most intense explosive eruptions with accompanying rapid lava effusion occurred between 25 and 29 November. Smaller infrequent explosions and extrusions continue through the present (June 2019). The delay between intense unrest and eruption caused considerable challenges to emergency responders, local and national governmental agencies, and the population of Bali near the volcano, including over 140,000 evacuees. This paper provides an overview of the volcanic activity at Mount Agung from the viewpoint of the volcano observatory and other scientists responding to the volcanic crisis. We discuss the volcanic activity as well as key data streams used to track it. We provide evidence that magma intruded into the mid-crust in early 2017, and again in August of that year, prior to intrusion of an inferred dike between Mount Agung and Batur Caldera that initiated an earthquake swarm in late September. We summarize efforts to forecast the behavior of the volcano, to quantify exclusion zones for evacuations, and to work with emergency responders and other government agencies to make decisions during a complex and tense volcanic crisis.

## Introduction

In August 2017, Mount Agung displayed increasing unrest, manifested as anomalous heat flow and felt earthquakes. The potential for activity at Agung was daunting for three primary reasons. First, this enormous ~3000-m-high volcano looms above the nearby landscape, threatening nearly 200,000 people who live within the hazard zone. Second, the previous eruption in 1963 killed over 1000 people^1^; with a Volcanic Explosivity Index (VEI) of 5, it was one of the ten largest volcanic eruptions of the 20^th^ Century. Third, lack of instrumental monitoring prior to previous eruptions meant that there was no geophysical monitoring basis for interpreting the somewhat unusual sequence of unrest. In this article, we document the volcanic unrest and eruption (still continuing), and provide a summary of actions taken by our group from the Indonesian Center for Volcanology and Geological Hazard Mitigation (CVGHM) and the USGS-USAID Volcano Disaster Assistance Program (VDAP), and by our colleagues, and other government officials in an effort to mitigate the impacts of any potential volcanic eruption.

## Background

Mount Agung is sacred to the Hindu population of Bali, and is a great tourist attraction on the island. The volcano also supports intense agriculture, as abundant cold springs issue on its south flank at elevations of 300–500 meters. The springs form the basis for the Balinese subak system of water distribution that supports irrigation of terraced rice paddies on the volcano’s lower slopes. Higher up, deposits of volcanic ash and lapilli are quarried for the aggregate needed to support development of urban growth near the Balinese capital city of Denpasar and the tourist destinations of Ubud and Kuta. Mount Agung clearly supports the region economically, but it also poses great threats, as was made apparent during the last eruption in 1963.

**Figure 1:**
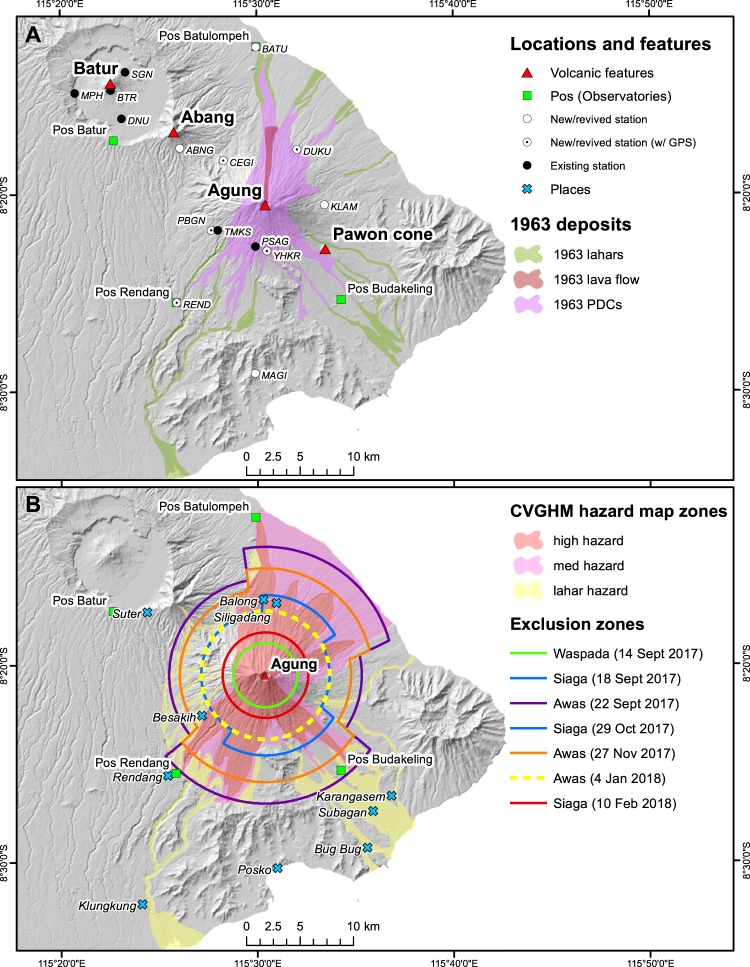
Maps of Agung. (**A**) Location map of the area surrounding Mount Agung, in eastern Bali. Volcanic features (red triangles), locations of the observatories (pos, green squares), and relevant towns (blue crosses) are overlain on a Shuttle Radar Topography Mission (SRTM) 30 m DEM. The 1963 lava flow, pyroclastic density current, and lahar deposits are modified from^2^. (**B)** CVGHM hazard zones from the published hazard map^39^ are shown along with the exclusion zones established through the crisis. Note that the 18 September and 29 October exclusion zones (in blue) are the same.

In that year, earthquakes were felt on 16 February and 17 February, followed soon by loud noises and explosions around 19 February^1^. Fire fountaining of lava within the summit crater resulted in accumulation of lava and eventual effusion through a low-point in the crater wall, traveling 7.5 km down the north flank. Pyroclastic density currents (PDCs) resulted in the eruption’s first fatalities only a day later^1,2^. On 17 March 1963, a month after the initial felt earthquakes, a VEI 5 paroxysm created a 19- to 26-km-high plume with associated 10.5 to 14-km-long pyroclastic density currents and follow-on hot and cold lahars, which together killed over 1000 people^1,2^ (Fig. 1A). Other deadly paroxysms occurred again in May, ejecting ballistics to 8 km^3^ and producing additional PDCs. Explosive activity continued until early 1964. Fatalities from ballistics occurred as far as 6.5 km^3^ from the summit. The range of magma compositions erupted varied from basaltic andesite to andesite, with relatively few phenocrysts, and a high sulfur release (7.0–7.5 Mt of SO_2_) was consistent with rapid rise of volatile-rich mafic magma^4^. Notably, in September of 1963, after ~40 years without eruption, basalt and basaltic andesite lava issued from a cone in the nearby Batur Caldera (Fig. 1), only 18 km NW of Mount Agung’s summit^5^. Batur remained active throughout the 1960s and 1970s, with its most recent eruption in 2000^6^.

In recent years, stratigraphic studies at Mount Agung have revealed an eruptive frequency of one VEI ≥ 2–3 eruption per century, and a dozen or so VEI 4 and 5 eruptions in the past 5000 years^7^. Satellite interferometry was interpreted to show inflation of the volcanic edifice in the period 2007-09^8^, which catalyzed our installation of new seismic and geodetic monitoring networks around the volcano in 2012 to be better prepared for future eruptions.

## Scientific Observations

### Seismicity

During the fifty years since the 1963 eruption, almost no local earthquakes were recorded on the CVGHM network at Mount Agung, and seismic energy was dominated by cultural noise from the south flank of the mountain. As of 2017, the seismic monitoring network consisted of two short-period stations on the south and southwest flanks of Mount Agung ~4 and 5 km from the summit and four short-period stations in the Batur Caldera (Fig. 1A). Throughout the crisis, the primary data streams used to monitor unrest were real-time seismic data from the CVGHM network and earthquake hypocenters from the Indonesian Meteorology, Climatology, and Geophysics authority (BMKG).

The CVGHM network was used to make visual observations, conduct daily earthquake counts, and compute RSAM (Real-time Seismic Amplitude Measurement). Although many hypocenters were also manually computed using the CVGHM network during the crisis, these were used primarily to verify and supplement BMKG solutions and were not consistently catalogued. The description of activity below is a brief summary of the observed seismicity from all data sources.

**Figure 2:**
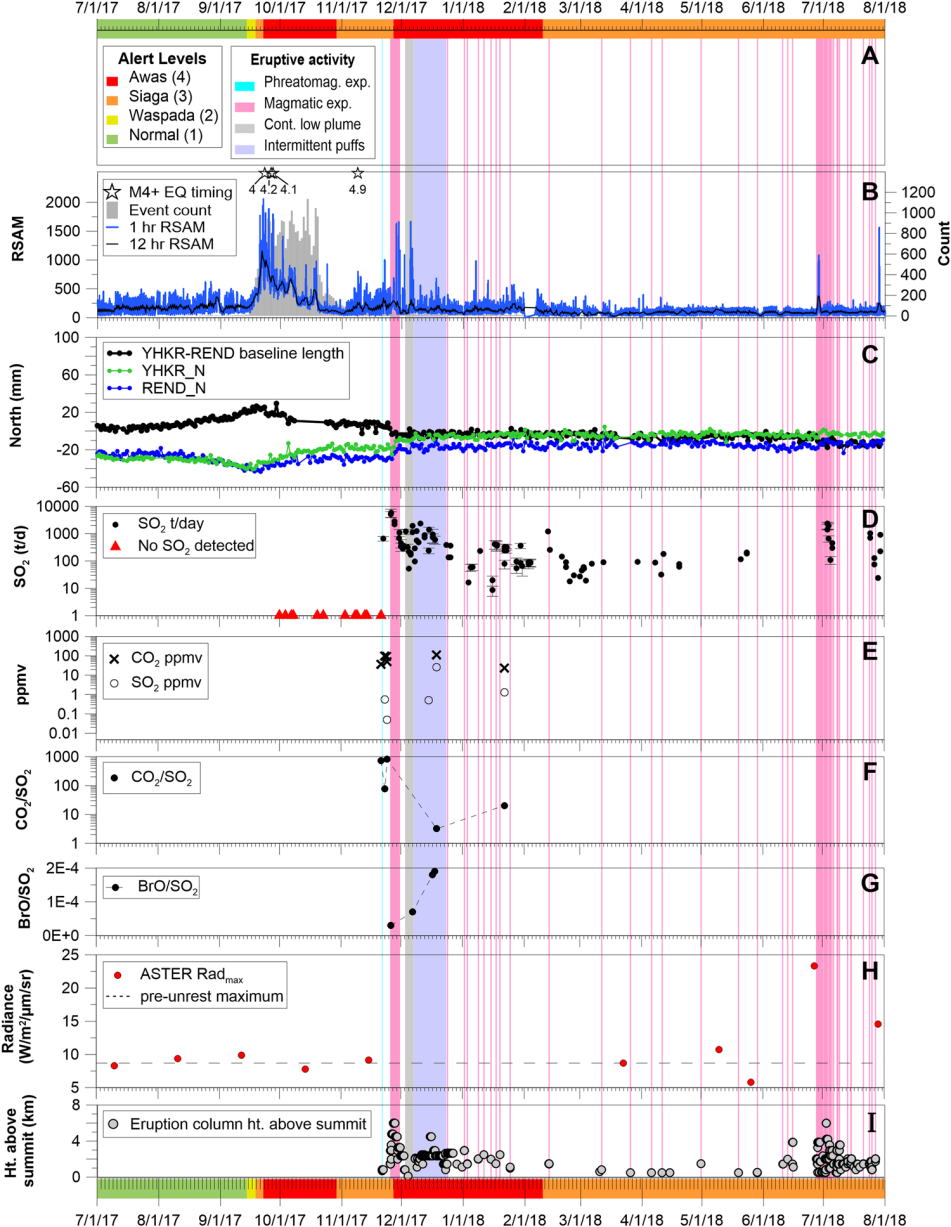
The timeline of the 2017–2018 unrest and eruption at Mount Agung, showing (from top to bottom) (**A**) Alert level changes; (**B**) RSAM from TMKS, and daily seismic event counts. Magnitude ≥4 earthquake times are displayed as labelled stars across the top of the panel. Note: the RSAM peak in late July 2018 is related to the large (M6.4) tectonic events near the island of Lombok; (**C**) GNSS displacements and baseline length between YHKR and REND (also known as RNDG) stations; (**D**) SO_2_ emission rates from ground-based mobile DOAS; **(E**) CO_2_ and SO_2_ mixing ratios above ambient background from drone-transported Multi-GAS; (**F**) CO_2_/SO_2_ ratios (molar) from Multi-GAS; (**G**) BrO/SO_2_ ratio from mobile DOAS; (**H**) Advanced Spaceborne Thermal Emission and Reflection Radiometer (ASTER) maximum radiance values from the crater, with pre-unrest maximum radiance (8.7 W/m^2^/μm/sr) plotted as dashed line (see also Supplemental Figures Fig. S1); and (**I**) eruption column heights (as measured above the 3.142 km summit). Running across the entire graph are phreatomagmatic (blue) and magmatic (pink) explosions, as well as periods of continuous ash venting (grey) and intermittent ash puffing (purple).

**Figure 3:**
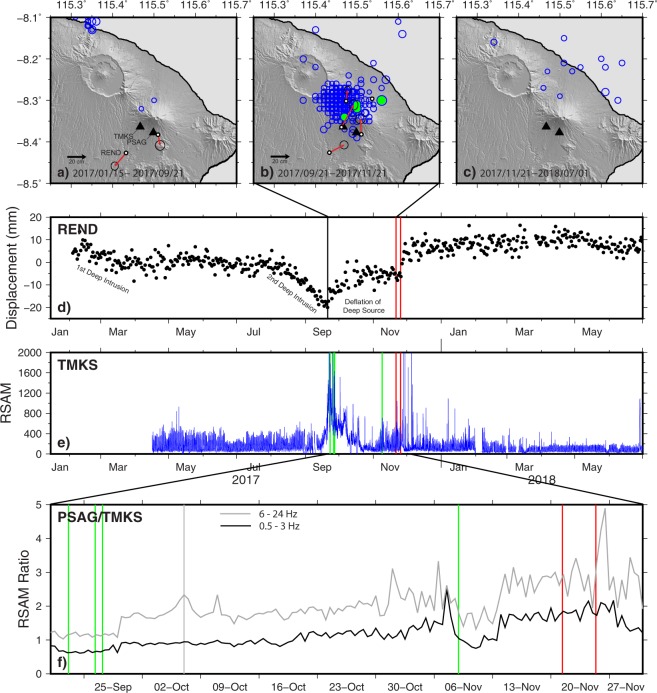
Regional BMKG Earthquake locations for (**A**) 2017/01/15 – 2017/09/21, (**B**) 2017/09/21 – 2017/11/21, and (**C**) 2017/11/21 – 2018/07/01. Earthquake circle size is scaled by magnitude (range M2.2 to M4.9). Locations are plotted from public data listed to two decimal places, accounting for the gridded appearance. M4+ events are colored green. GPS displacement vectors (small circle is station location: larger circle is approximate error ellipse) demonstrate (**A**) movement away from the volcano during deep inflation and (**B**) movement to the N and NE as a result of a combination of dike intrusion and deflation of a deeper source. No clear deformation source was seen in (**C**). (**D**) Detailed GPS time-series and (**E**) RSAM data (1 hour) for stations REND (North) and TMKS, respectively. (**F**) Frequency filtered RSAM (12 hour) ratios between seismic stations PSAG and TMKS, the two closest stations (4.0 and 5.0 km, respectively) to the Agung summit, which were operating continuously both before and during the seismic crisis. Both instruments are Mark Products L4 seismometers with a one-second period. The frequency bands 0.5–3 Hz (black) and 6–24 Hz (gray) are shown in order to remove a persistent cultural noise source at ~4–5 Hz. Both bands show a general increase in ratio over time approaching the eruption, after which the ratio began to decrease. Green lines in (**E**),(**F**) represent times of M4+ earthquakes shown in (**B**). Red lines in (**D**)–(**F**) show timing of the phreatomagmatic eruption onset (21 November) and onset of larger explosions (25 November). The gray line in (**F**) shows the timing of a large steam emission visible at the summit on 7 October. The abrupt changes prior to this on 29 September are due to changes in analog telemetry. See text and Fig. 1 for other details.

A swarm of earthquakes (M2.3-3.9) was recorded in mid-May 2017, located NW of the Batur caldera, with a maximum reported intensity of MMI III. After several months of gradual increases, earthquake rates and seismic energy increased rapidly between 16 and 22 September 2017 from tens of earthquakes per day to hundreds of earthquakes per day (Fig. 2). Felt reports and seismic-wave-arrival times on local stations suggested that the observed volcano-tectonic (VT) earthquakes were located between Mount Agung and Batur Caldera (i.e., NW of Agung). However, regional hypocenter solutions produced by BMKG initially suggested that the events were closer to Mount Agung (Fig. 3). Seismicity peaked on 22 September with >800 earthquakes of magnitude >1 recorded by the CVGHM seismic network (Fig. 2B). Earthquake magnitudes also increased, with a M4.2 (BMKG) that occurred on 26 September. These earthquakes were all high-frequency, VT earthquakes.

VT event rates decreased significantly on 20 October (Fig. 2B) and continued to decrease through early November. During October and November 2017, we augmented the seismic monitoring network by adding six broadband digital stations and one short-period digital station at sites near the mountain to improve detections and locations across the network (Fig. 1A).

In late October, earthquake hypocenters began to spread to the N and NE of Mount Agung while continuing to occur to the NW. By early November, earthquake rates had dropped to steady levels of ~300 earthquakes per day with large M3+ events still common. While earthquake rates decreased during this time period, RSAM ratios of the closest two stations showed an indication of magma migration toward the summit crater and RSAM values showed a subtle but persistent long-term trend increase, a trend that continued into the initial phreatomagmatic eruptions in late November (Fig. 3). On 8 November, 2017 ~22:00 UTC, BMKG recorded a M4.9 and a series of aftershocks located ~10 km NE of Mount Agung (Fig. 3). Shortly afterwards, small, low-frequency (LF) and VT earthquakes proximal to the summit were noted regularly. The first clear signs of tremor (~40–120 second duration; broadband 1–10 Hz) were recorded by the early hours of 12 November UTC. In retrospect, by this time, magma was clearly invading the upper levels (<5 km) of the Mount Agung edifice. VT and LF earthquakes continued at low rates and RSAM values gradually increased through the first phreatomagmatic eruption on 21 November, but the eruption itself was not recorded seismically. More tremor was recorded a day after the 21 November phreatomagmatic eruption, and VT and LF event rates continued at low levels. The onset of magmatic eruption was preceded by a swarm of 22 larger LF earthquakes on the morning of November 25 local time, although the onset of lava effusion, which was first detected in satellite data the same day, was not recorded seismically.

After the onset of effusion, earthquake rates and RSAM values continued at pre-eruptive levels until a significant increase on 8 December. Fluctuations in seismicity were not correlated with changes in visual observations of eruptive activity at this time. Although Mount Agung began producing regular, discrete explosions early on in the effusive phase, none of the explosions were recorded seismically on the CVGHM network until 23 December. After this date, almost all explosions at Agung were recorded on the CVGHM seismic network. Prior to each explosion, however, earthquake rate or energy increases were either absent or, in some cases, too subtle to reliably forecast subsequent explosions. Starting after the first lava extrusion on or just before 25 November, tremor episodes lasting 30–90 minutes occurred sporadically, but were not typically correlated with eruptive behavior. There is convincing evidence (repeated occurrence during afternoon rains, relatively high frequency content, and visual observations of rain clouds at the summit) that suggests these episodes were related to rainfall at the summit, plausibly due to interaction of rainfall with scalding rock by means of growing cracks in the crater lava. During the most intense phase of the eruption, transit of lahars was seismically recorded on the N and S flank of the volcano. These lahars were thought to have originated by rainfall on ash that was deposited on the upper flank of the volcano during the initial explosive activity during the period of approximately 21–30 November.

After the most intense phase of eruptive activity in late November, seismicity decreased. Though rate increases in LF seismicity culminated in Strombolian-type explosions on 19 January 2018, and large (M3+) VTs continued in February and March, overall earthquake rates decreased to tens of events per day or fewer. On 23 June 2018, a small swarm of VT and LF seismicity began and increased until an explosion on 27 June and additional lava extrusion and ash emissions on 28–29 June, which was accompanied by monochromatic tremor. On 2 July 2018, Strombolian activity was recorded as a series of seismic explosion signals. Seismicity associated with intermittent explosive activity continued through the present (June 2019).

### Deformation

Deformation of Mount Agung is monitored by a network of 5 continuous GNSS stations (Fig. 1A) that was installed in 2012. By 2014, all of the sites had ceased transmitting data, but they were revived in late 2017, and some data extending back to 2016 were recovered. Surface displacements preceding and accompanying 2017–2018 eruptive activity occurred in several discrete episodes, as exemplified by the time series from station REND (Figs 2C and 3D located ~12 km south-southwest of the volcano’s summit. Prior to the onset of the seismic swarm in mid-September, two periods of apparent inflation were evident, in February–March 2017 and again during August–September 2017. During both periods, motion of operational stations was away from Agung (Fig. 3A), with the later inflationary epoch being the larger of the two (for instance, southward motion of REND was ~5 mm in February–March and ~20 mm in August–September). The first episode was not accompanied by seismicity. The second was accompanied by slowly increasing seismicity, and no significant deformation occurred during the intervening months. A simple Mogi model^9^ of the displacements suggests a pressure increase at 10–20 km depth, although the few data points do not permit a more detailed assessment. The deformation is not apparent in InSAR data spanning the time period, probably due to the small magnitude of the displacements^10^.

The rapid increase in seismicity in September was accompanied by a significant change in deformation at all sites (Fig. 3B). Station REND, for example, began moving north towards the volcano’s summit. InSAR results spanning September–October suggest the emplacement of a dike at ~10 km depth between Agung and Batur^10^ while GNSS stations—particularly REND—are consistent with a combination of dike intrusion to the northwest of Mount Agung and deflation of a deeper source (the same source that inflated in February–March and August–September). A co-eruptive episode of deformation in November 2017 coincided with the onset of lava extrusion and is consistent with deflation of a source beneath Mount Agung, although the data cannot distinguish the depth of this source. From mid-December 2017 through April 2018, surface deformation was minor. From May to mid-June 2018 shallow inflation was detected, followed by extrusion of lava and an increase in explosion frequency in late-June to July 2018.

### Remote sensing and ash samples

**Figure 4:**
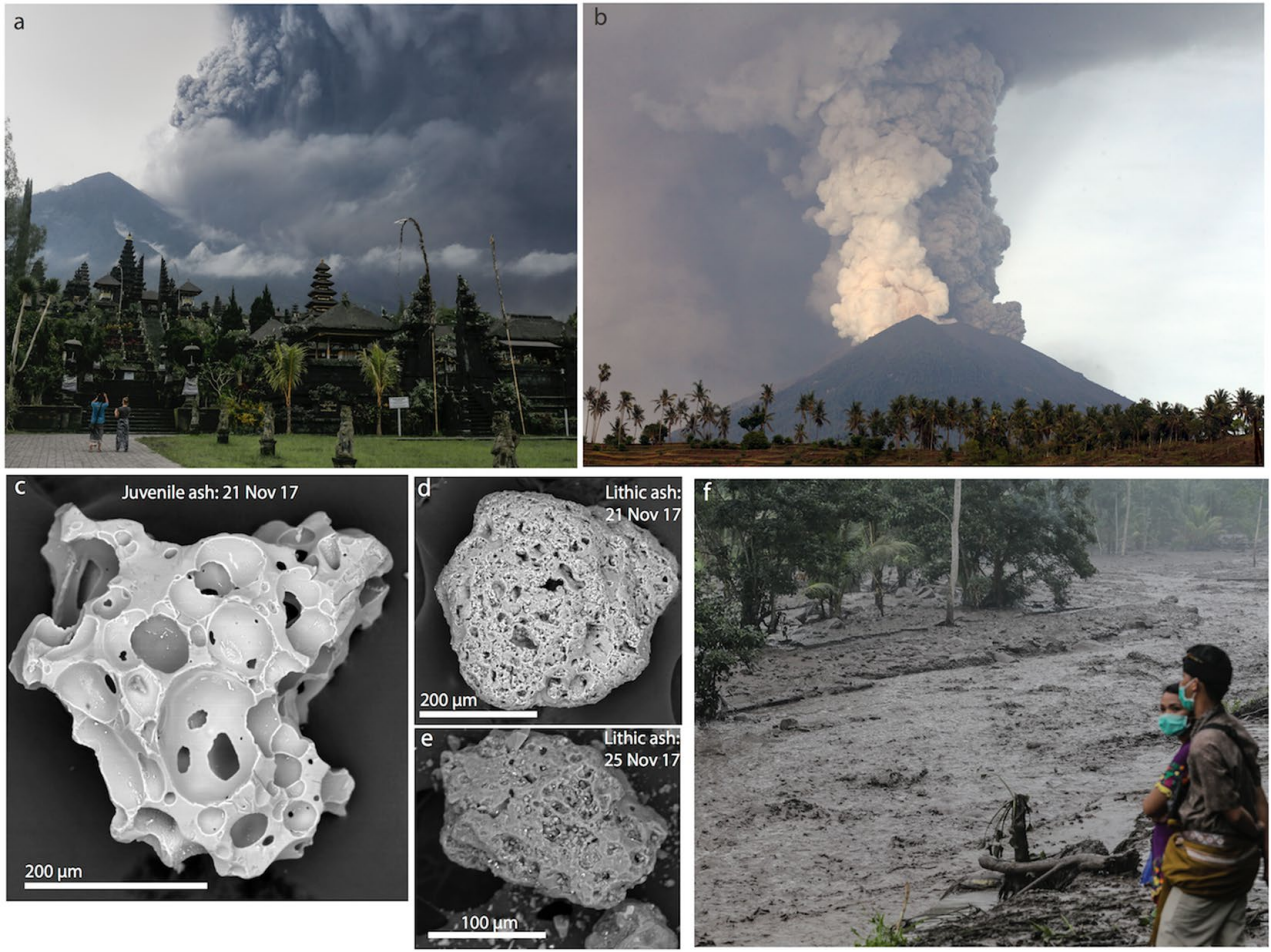
Images from the November volcanic eruptions. (**A**) Looking northeast from Besakih Temple during eruption on 26 November 2017. Photo by Johannes P. Christo. (**B**) View east toward Mt. Agung on 27 November 2017 from Culik marketplace. Dark ash-rich and white steam-rich plumes emerge simultaneously. Photo by Firdia Lisnawati. (**C**) Juvenile scoria fragment erupted on 21 November 2017. (**D**,**E)** Lithic fragments erupted on 21 November and 25 November, respectively. (**F**) Lahar on 28 November 2017 at Tukad Yeh Sah river. Photo by Johannes P. Christo.

**Figure 5:**
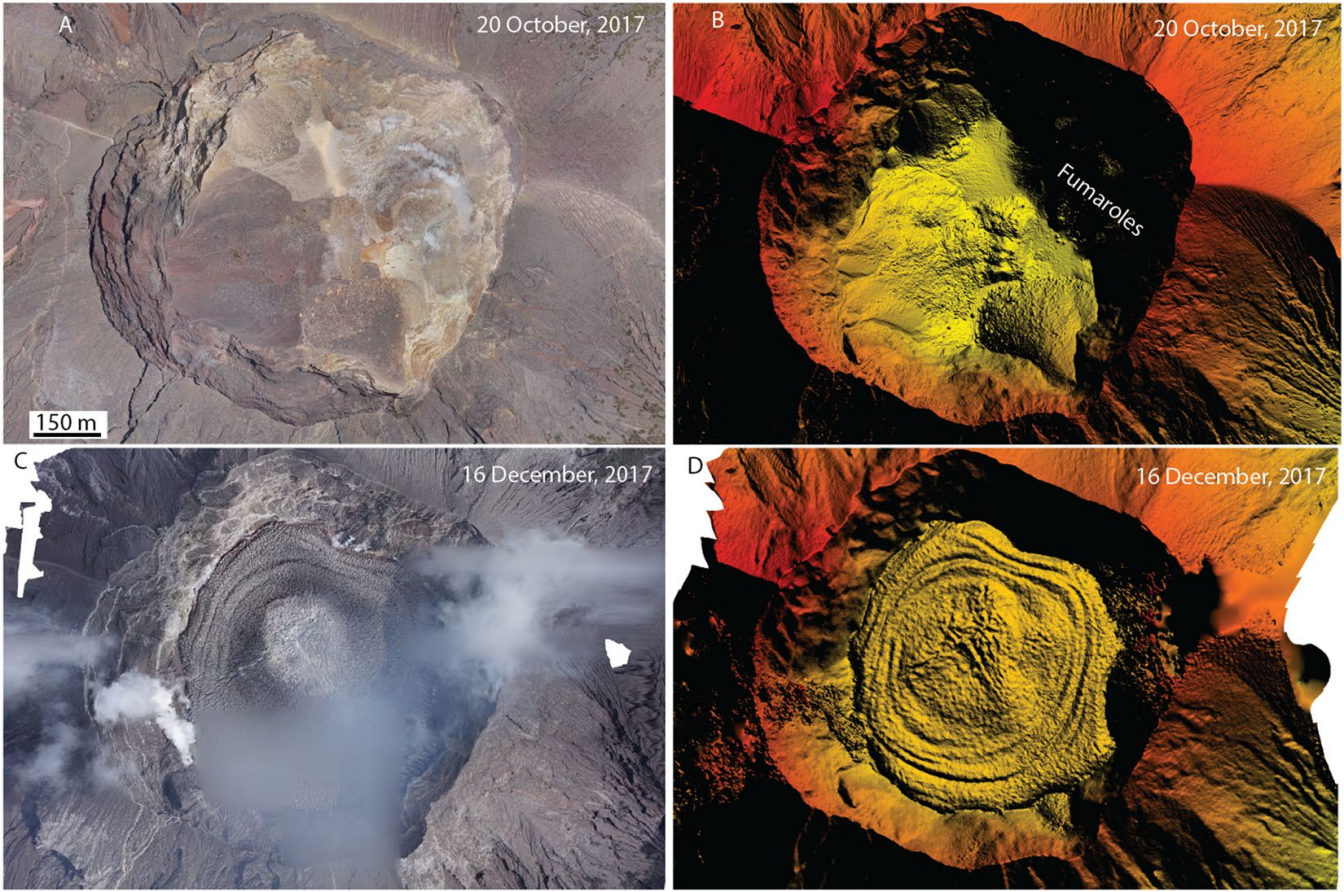
Images captured by drone flights over Mt. Agung crater on 20 October 2017 and 16 December 2017. (**A**) Rectified photo montage of pre-eruption conditions with steaming in the eastern wall. (**B**) Hillshade digital elevation model with false colors showing relative elevation (yellow to red). (**C**) Post-eruption photo montage that shows the lava flow. (**D**) Hillshade digital elevation model as in (**B**), where the lava flow contains concentric pressure ridges created during outward flow from the central vent. Cracks are visible propagating from the center vent region.

Satellite data provided frequent views of Mount Agung’s summit crater and edifice. Steaming in the crater was first reported in September 2017. High resolution satellite data showed that steaming had been intermittently visible since at least September 2016. Satellite data document increases in the volume and area of steaming and episodic ponding of water that emanated from a talus pile near the base of the NE crater wall beginning as early as 14 September 2017. After the first explosive activity on 21 November, satellite data detected a new 100-m-diameter crater centered in the larger summit crater that served as the conduit for subsequent eruptions. Ash samples from the 21 November event include minor juvenile components, but are dominated by remobilized edifice lithic material (Fig. 4D,E). Collected bulk ash samples were analyzed for their major-element chemistry and had bulk chemistry of andesite. Sequential sampling revealed an apparent increase from 55 to 59 wt.% SiO_2_ in bulk composition of the erupted ash from 22 November 2018 to 29 November 2018. Semi-quantitative analyses of juvenile glass confirmed an andesitic composition. A small lava flow was first observed within this crater on 25 November and by 27 November had covered the crater floor (Fig. 5). When lava effusion slowed significantly, less than a week later, the lava flow had covered the floor of the crater and reached a maximum thickness of about 121 m and a volume of about 24 million m^3^. At this point, the lava had reached about one-third of the height of the low point in the crater wall, located along the south rim. By 5 December 2017, following a one-week pause in activity, new fractures had begun to form over the central part of the lava flow. As the fractures grew wider, imagery suggested that molten lava had flowed in from below to seal the fractures. Over the next several months, explosions continued to modify the lava surface, creating new explosion pits and depositing coarse eruption debris on the lava surface. Localized inflation of the central vent area surface was observed shortly before one of the explosions. Satellite imagery revealed that a new period of lava extrusion, which began on 28 June 2018, produced new material that covered nearly the entire November crater lava flow and added an additional ~10 m to its thickness.

### Gas composition and emission rate

Due to the previous lack of long-lived fumaroles at Agung since its 1963 eruption, no geochemical monitoring program or instruments were in place prior to the 2017 unrest. Conditions near the summit were considered too hazardous for proximal sampling, so regular attempts to measure sulfur dioxide (SO_2_) using ground-based remote sensing techniques began in October 2017 after steam emissions had visibly increased. Despite the presence of a small, persistent plume and reports of sulfurous-smelling gases from unauthorized hikers, 12 mobile DOAS (Differential Optical Absorption Spectrometry)^11^ campaigns made between 1 October and 14 November 2017 all failed to detect SO_2_.

**Figure 6:**
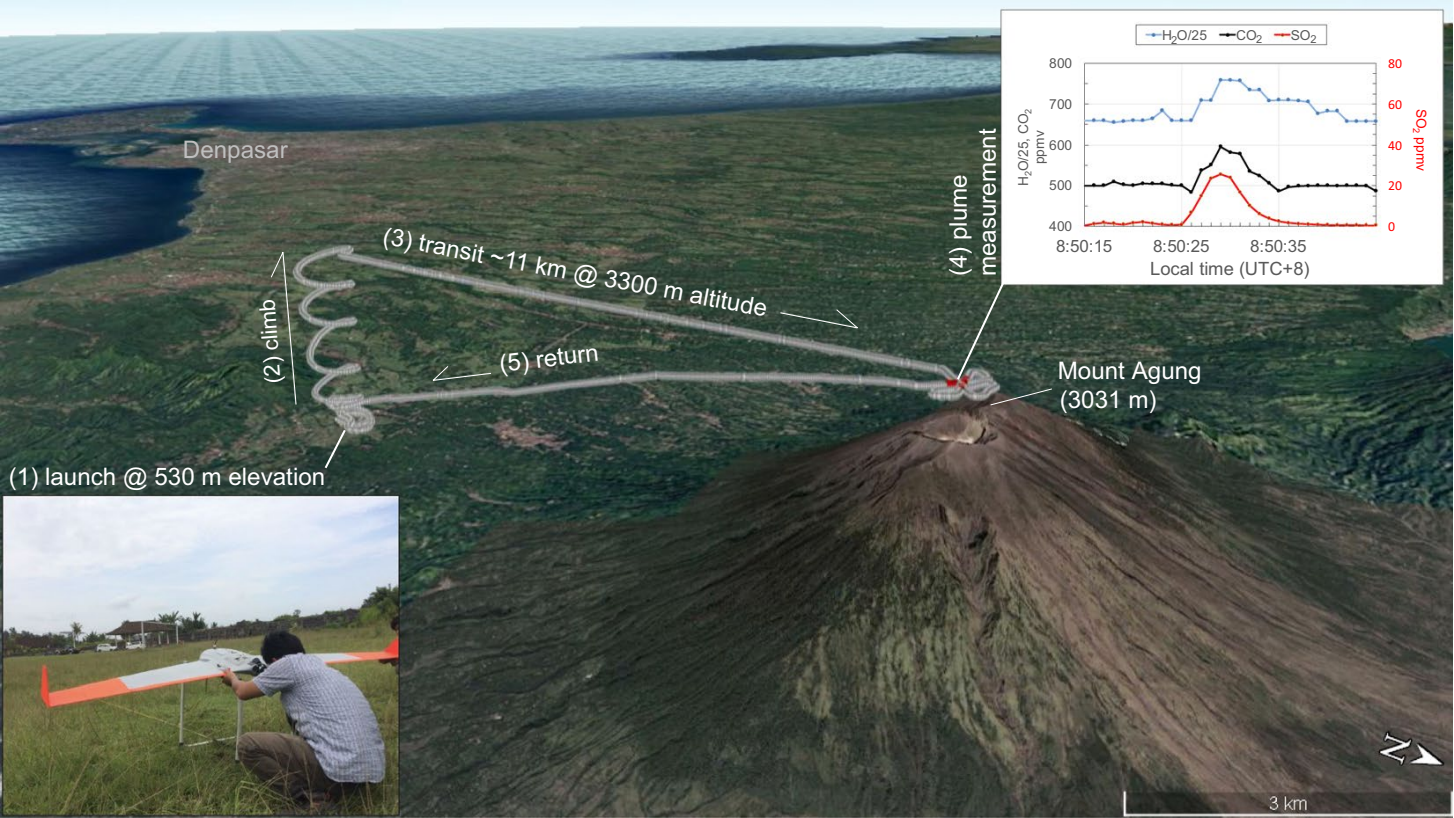
Perspective digital elevation model (from Google Earth) that displays drone flight path on 19 December 2017 from Rendang region near the Agung observatory Pos (1), followed by direct spiral ascent (2), transit to Mount Agung (3), plume measurements (4), and return (5). Inset at bottom left shows the Ai450 drone model Aeroterrascan. The inset in upper right displays the multi-GAS H_2_O/25 and CO_2_ signals on the left ordinate, and SO_2_ on the right ordinate. The plume was intersected over a ten-second interval centered at 8:50:30 local time. Google Earth imagery from Landsat/Copernicus collected on 16 September 2017 and 30 December 2016.

In mid-November, we pioneered the use of a fixed-wing drone (AeroTerraScan model Ai450) instrumented with a miniaturized multi-GAS^12,13^ (Multiple Gas Analyzer System) to obtain airborne *in situ* measurements of plume H_2_O vapor, CO_2_, SO_2_, and H_2_S. The drone was launched from 530 m elevation at a location 11 km south of the summit and climbed to ~3,300 m for sampling (Fig. 6). The first successful measurements were obtained at 00:21(UTC, 08:21 local time) on 21 November and revealed a large plume-related CO_2_ anomaly (ΔCO_2_ = 36 ppmv; “∆” indicates that the measurements are reported with ambient background subtracted); SO_2_ was below the sensor detection limit (~0.05 ppmv; Fig. 2E). While no prior baseline gas measurements were available for comparison, airborne measurement of in-plume CO_2_ anomalies of this magnitude are uncommon^12,14–17^ and these data were viewed as a significant indication of unrest. Approximately 9 hours later, the first phreatomagmatic explosion occurred. Ground-based DOAS measurements the following day (22 November) yielded an SO_2_ emission rate of 660 t/d (Fig. 2D). Three different drone flights on 23 and 24 November found large CO_2_ anomalies (ΔCO_2_ = 49–98 ppmv), very low SO_2_ mixing ratios (SO_2, MAX_ = 0.55 ppmv on 23 November; 0.05 ppmv on 24 November), and trace H_2_S (<0.17 ppmv on 24 November. These data showed that gas emissions were very CO_2_-rich and S-poor, and average molar CO_2_/SO_2_ ratios increased dramatically from 77 to 824 on 23–24 November prior to the start of the main magmatic explosive phase at 9:20 UTC on 25 November (Fig. 2F).

The highest SO_2_ emission rate was measured on 26 November (5,500 t/d) but quickly fell to 180 t/d by 1 December. Gas emissions during lava effusion in December were highly variable (SO_2_ = 140–1500 t/d; monthly median = 390 t/d, n = 14) and magmatic in character; a drone flight on 19 December intercepted a dense plume with clear H_2_O, CO_2_, and SO_2_ peaks (H_2_O/CO_2_ = 21, CO_2_/SO_2_ = 3.2; SO_2, MAX_ = 26.1 ppmv). The DOAS measurements picked up very low levels of BrO in the large 26 November plume (BrO/SO_2_ = 3E-5). Subsequent data showed an increasing trend up to BrO/SO_2_ = 1.8 and 1.9E-4 detected on 17 and 18 December, respectively (Fig. 2G). The increasing BrO/SO_2_ ratios are consistent with increased degassing of shallow magma from the growing lava flow in the crater releasing HBr, followed by reactions in the atmosphere partially converting HBr to BrO^18^. Further DOAS measurements in January and February showed that SO_2_ emissions were decreasing (median January SO_2_ = 230 t/d, n = 12; February = 220 t/d, n = 4). SO_2_ emissions briefly jumped to more than 1000 t/d in the week after the 28 June 2018 extrusion event, but then quickly returned to low baseline values (<200 t/d) by the beginning of August 2018.

### Summary of Basic Timeline

Below, we provide a timeline of events as they occurred, though in some cases, as with deformation, they were not detected at the time. We frame the timeline in terms of alert-level changes so that the reader can appreciate the events and reasoning that led to those changes. The date of alert-level change is denoted in the header for each entry, though key events and observations begin prior to and after that date.

*14 September 2017–– Upgrade to Level 2:* The first swarm of earthquakes was recorded by the local Agung and Batur seismic network in mid-May 2017. Figure 2 presents a timeline of observational and geophysical measurements from July 2017 to August 2018. By mid-July 2017—around the time that a small thermal anomaly was detected (Fig. 2H, Supplemental Figures Fig. S1)—RSAM values at Agung had deviated from baseline levels (Fig. 2B), and by mid-August, VT earthquakes were occurring daily, increasing significantly in September. In retrospect, we now know that a second episode of inflation was detected by GNSS from August-September, as well as by InSAR^10^. Unusual fumarolic activity in the northeast part of the summit crater, along with increasing seismicity, prompted an alert level change to Waspada (Level 2) on 14 September (Table 1, Figs. 1B and 2A).

**Table 1:** Alert level changes, observations, exclusion zones and outcomes.

Date	Alert Level	Observations leading to alert level change	Observations leading to retaining alert level	Exclusion zone^*^	Outcomes
9/14/2017	Waspada (2)	$\cdot$ thermal anomalies $\cdot$ fumarolic activity $\cdot$ increase in VT seismicity		3 km	
9/18/2017	Siaga (3)	$\cdot$ increase in fumarolic activity $\cdot$ increase in felt earthquake freq. and M $\cdot$ increase in VT seismicity		6/7.5 km	
9/22/2017	Awas (4)	$\cdot$ increase in fumarolic activity $\cdot$ increase in thermal anomaly sz. $\cdot$ peak in seismicity $\cdot$ increase in felt seismicity $\cdot$ event tree on 9/20/2017 $\cdot$ modeling showed that large PDCs could travel >10 km in <3 minutes; and that a VEI 3 eruption could result in 1.6 m of ash within 15 km $\cdot$ high disaster potential; many local people had no memory of 1963 eruption	$\cdot$ VTs closer to the summit $\cdot$ appearance of LFs $\cdot$ increasing felt seismicity (M4.3 on 9/27/2017) $\cdot$ event trees on 9/23, 10/02 and 10/17/2017	9/12 km	$\cdot$ ~140k people evacuated (inc. ~70k self-evacuated from outside the exclusion zone) $\cdot$ protective eyewear and masks recommended $\cdot$ communication network established through WhatsApp groups and radio $\cdot$ funding made available for evacuation expenses
10/29/2017	Siaga (3)	$\cdot$ decrease, followed by waxing and waning seismicity $\cdot$ lack of further lg. changes in deformation or to the crater		6/7.5 km	
11/26/2017	Awas (4)	$\cdot$ explosions began 11/21/2017 $\cdot$ magmatic explosions began 11/25/2017	$\cdot$ cont. explosions $\cdot$ cont. lava flow effusion $\cdot$ open system thought to make forecasting difficult $\cdot$ high CO_2_ and SO_2_ $\cdot$ ashfall increased lahar risk	8/10 km	
1/4/2018	Awas (4)^†^	$\cdot$ decrease in explosion frequency		6 km	
2/10/2018	Siaga (3)	$\cdot$ decrease in explosion frequency $\cdot$ decrease in seismicity $\cdot$ decrease in SO_2_ emissions $\cdot$ decrease in thermal anomaly		4 km	

^*^Greater exclusion zone distances given for N, SE, SW sectors.

^†^Alert level remained the same, but the exclusion zone limit was reduced

*18 September 2017–– Upgrade to Level 3*: Water ponding (possibly expelled from the edifice or alternatively condensed from fumaroles) was noted in the crater on 14 September and formed small deltas in the vicinity of the fumaroles. Increasing fumarolic activity, a growing thermal anomaly in the crater, and felt earthquakes (M3+) increased the level of concern of local populations. Rapidly increasing seismicity prompted an alert level change to Siaga (Level 3) on 18 September.

*22 September 2017–– Upgrade to Level 4*: Seismicity continued to accelerate rapidly and RSAM values peaked on 22 September (Figs 2B and 3), prompting another alert level change. In retrospect, we know there was also a change in the relative motion of GNSS-stations (Figs 2C and 3). GNSS stations south of the volcano registered movement toward the volcano, while a station to the northwest (CEGI) registered movement away from the volcano. The change to Level 4 (Awas) triggered evacuations. RSAM values then declined, but elevated seismic event rates, including large magnitude earthquakes (up to M4.2), persisted. On 7 October, a notable white-colored gas plume rose from the northeast crater floor ~1500 m above the summit crater, lasted for about an hour, and was detected seismically (Fig. 3F). This was the tallest plume observed before the eruption. Unauthorized climbers reported sulfur smells, rumbling noises, and fumarolic activity from the northeast crater floor. However, SO_2_ emissions were below the detection limit as measured by mobile DOAS at 12 km distance (Fig. 2E).

*29 October 2017–– Downgrade to Level 3:* Seismic event rates declined sharply on 20 October, though VTs started to move closer to the summit (proximal events). With the decrease in seismic event rates and the long (one-month) duration of evacuations, the alert level was lowered to Siaga (Level 3) on 29 October. In early November, RSAM values began to increase slowly (Figs 2B and 3). On 8 November, an M4.9 earthquake was recorded and was felt by people (Modified Mercalli Intensity, MMI II–V) as far as ~60 km from the volcano. This was the largest recorded VT event during the crisis period (Figs 2B and 3).

*26 November 2017–– Upgrade to Level 4:* In mid-November, LF events and tremor appeared, and seismic event locations moved closer to the volcano. Drone flights fitted with a multi-GAS above the volcano’s crater detected a CO_2_-rich plume early on 21 November (Fig. 2E,F). The 2017 Agung eruption began with a small phreatomagmatic explosion on 21 November 9:05 UTC, with ash emissions to 700 m above the summit (Figs. 2I and 4). A moderate amount of SO_2_ (660 t/d) was detected the following day by mobile DOAS, consistent with magma degassing (Fig. 2D). Multi-GAS drone flights detected elevated levels of CO_2_ on 23-24 November (Fig. 2F). Larger, continuous explosions began on 25 November at 9:20 UTC and satellite observations detected the presence of a lava flow within the crater. The ash column reached ~6 km above the summit (~9 km asl) by 26 November (Fig. 2I) and traveled ESE resulting in closure of the Praya airport in Lombok (~95 km SE of Agung crater) on 26–27, 30 November and 1 December. On 26 November 23:00 UTC, the alert level was raised to Awas (Level 4). The tropical cyclone Cempaka changed the wind directions, and pulled the ash cloud south and west, forcing closure of the Denpasar’s Ngurah Rai airport (~60 km SW of Agung crater) during 26–29 November. High SO_2_ emissions were detected by mobile DOAS and the OMI (Ozone Monitoring Instrument) satellite. Lightning, loud rumblings, and lahars were produced (Fig. 4F) as a result of rainfall mobilizing ash deposits from late November. Two plumes were emitted on 26–27 November (Fig. 4B), with a dark, ash-rich part emanating from the main crater, and an abundant white vapor plume coming from the former fumarole field. By 27 November, lava covered the crater floor (Fig. 5C,D) and began to rapidly fill the summit crater, until slowing on 29 November; plume heights then declined as well. Some ash was deposited around the volcano: it was thicker and extended further in the WSW direction in line with the prevailing wind direction during the largest ash emissions period. Rainfall-induced lahars were generated within 16 drainages on the NNW, N, ENE, SE, S and SW parts of the volcano in late November, with the most significant flow the Tukad Yeh Unda river on the SW flank down to the Badung strait (~30 km from the Agung summit). The continuous explosive period was followed by a semi-continuous, low-level plume until 4 December, when a period of frequent (every 30–60 min), aseismic, ash ‘puffs’ (vapor plumes) began (Fig. 2).

*10 February 2018–– Downgrade to Level 3:* Around 23 December 2017, the regular puffing ceased and daily to weekly, discrete, seismically-detected explosions began (pink vertical lines in Fig. 2), producing plumes typically up to 2.5 km above the summit (~5.5 km asl) and leaving explosion pits in the cooling lava flow. As explosion frequency ceased, the exclusion zone was reduced to a radius of 6 km on 4 January. Minor Strombolian explosive activity was observed on 19 January 2018, after which the frequency of explosions declined significantly. The alert level was lowered to Siaga (Level 3) on 10 February. Between February and late June, there were intermittent discrete explosions, and generally low (but above background) SO_2_ emissions and seismicity rates (Fig. 2). A swarm of VT events on 23 June 2018 preceded a small explosion on 27 June 2018 and was followed by lava extrusion and continuous ash emission on 28–29 June 2018. The continuous ash emission to the WSW affected flight operations at the Denpasar, Bali and Jember, East Java airports on 28–29 June 2018 (UTC). At 13:04 (UTC) on 2 July, a Strombolian eruption threw incandescent material as far as 2–3 km from the summit crater. Even though the exclusion zone had been set to a radius of 4 km, thousands of people outside this zone self-evacuated due to the fear that incandescent material would travel farther, and due to the loud thundering sounds produced by the volcano. Ash emissions from these explosions moved west, causing airport closures in East Java (Banyuwangi and Jember) on 3 July. Afterwards, there was a period of numerous small explosions, gradually declining in frequency through July 2018 (Fig. 2). Minor seismicity continued. On 29 July and 5 August, two large earthquakes of M6.4 and M6.8 hit N of Lombok island (<120 km E of Mount Agung). Continuous degassing of a thin white plume was observed following these earthquakes; however, no other changes in eruptive activity were observed directly following these earthquakes; instead, similar low-level explosions continue through the time of this writing.

## Scientific Interpretation and Forecasting

### Volcano Model

**Figure 7:**
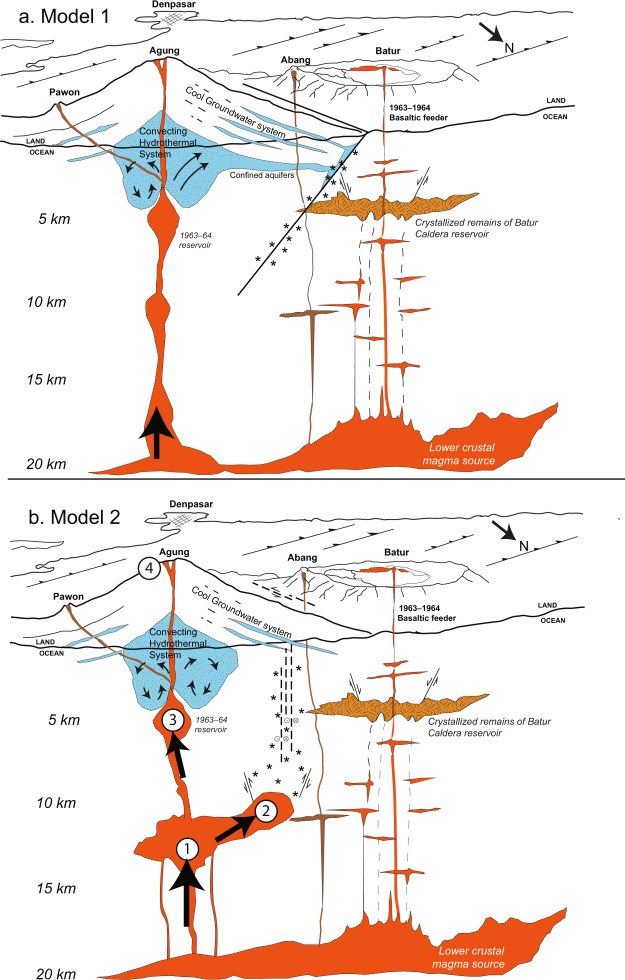
Conceptual cross-sections representing the magmatic plumbing system along a line parallel to the volcanic vents of Pawon, Agung, Abang and the post-caldera Batur cone. The section trends along a 300-degree compass direction and is projected onto a surface cut parallel to the north coast of Bali island. Because this direction is parallel to the inferred dike, its extent in this 2D section is not representative of dike width or volume. Surface features are based on a Google Earth perspective version of the digital elevation model of eastern Bali. Thrust faults shown on the surface are hypothetical; they are inferred from the structural model of McCaffrey and Nabelek (1987)^42^. (**A**) Conceptual cross-section created and used for reference during the volcanic crisis of Agung during October 2017 – January 2018, in which we speculated that magma rising beneath Agung volcano pressurized confined aquifers which in turn activated a pre-stressed fault located between Agung volcano and Batur caldera, resulting in volcano-tectonic earthquakes^21^. (**B**) Conceptual cross-section showing our current model of magma ascent beneath Agung. This model includes deep intrusion beneath the Agung edifice and a 300°-trending dike beneath the region between Mount Agung and Batur caldera. Presence of this intrusive dike is based on the interpretation of InSAR data that became available to the CVGHM and USGS response team in February 2018^10^. According to this model, dike intrusion caused uplift as well as the VT earthquake swarms. Fault geometry is inferred based on strike-slip focal mechanisms from earthquake swarms, although some thrust mechanisms along E-W oriented faults were also present. Numbers correspond to stages of magma ascent, as numbered in the discussion section.

As the volcanic crisis at Agung unfolded, a conceptual model of magma storage and ascent was developed to better frame forecasts; that model evolved as new data became available. Initiation of seismicity below Mount Abang, between Mount Agung and Batur caldera, led us to review the eruptive history and geologic context of the area. The temporal correlation between eruptions of Mount Agung and Batur caldera in 1963^5^ and their NW-SE alignment with Mount Abang (Fig. 1) raised the possibility of a subsurface connection between these volcanoes. In addition, seismic^19^ and petrologic^20^ data suggest that magma accumulates beneath both Mount Agung and Batur caldera at ~14–20 km depths, further raising the possibility of a deep magmatic connection between the volcanoes. However, several factors led us to favor a model of intrusion beneath Mount Agung. First, initial fumarolic activity and water ponding (possibly due to expulsion or to condensation from fumaroles) was limited to the Mount Agung crater, and there was no evidence for changes at Mount Abang or Batur caldera (gas emissions were minimal and there were no observed changes in the chemistry of the lake water in the Batur caldera lake). Perhaps an intrusion beneath Mount Agung had seismically activated faults NW of the summit through pressurization of groundwater (Fig. 7A)? Such a model is consistent with observations of VTs located along faults distal to vents at many other volcanoes (i.e., “distal VT” earthquakes^21^, where pressurization of aquifers is a likely mechanism^22^. The eventual eruption at Agung in November 2017 was therefore initially considered to be consistent with this hypothesis and conceptual model.

However, the best-fit source models for InSAR deformation data^10^ became available in mid-February 2018, and helped us to refine this picture (Fig. 7B). In this model, inflation NW of Mount Agung in late September – early October of 2017 is best fit by intrusion of a magmatic dike at ~10 km depth northwest of Mount Agung. This model of dike intrusion^10^ would further imply that pre-eruptive seismicity was dominated by events local to the magmatic intrusion, although pressurization of groundwater or magmatic fluids in the region above the inferred dike could have played a role in triggering earthquakes (Figs 3 and 7B). The primary deformation sources at Mount Agung during 2017–2018 thus include a pressure source—probably a subvolcanic magma reservoir—12 to >15 km beneath Agung that experienced both pre-eruptive inflation and deflation, as well as a phase of shallower dike intrusion to the northwest of the summit (mostly evident from InSAR) during late September–late October 2017. We further interpret the final stages of magma ascent and eruption using volcanic gas data. When the summit plume was first successfully surveyed by multi-GAS, nine hours prior to the first phreatomagmatic eruption on 21 November, SO_2_ was below detection, but CO_2_ was abundant, such that CO_2_/SO_2_ > 700. Such a high ratio could be due to very deep degassing (>10 km), or due to removal (scrubbing) of SO_2_ during magma ascent, such that originally CO_2_- and SO_2_-rich gas was depleted in SO_2_ before reaching the surface^23^. For magma to ascend 10 km in the 9 hours between gas measurement and eruption requires rapid ascent at >30 cm/s (0.01 MPa/s). These rates are commonly associated with large explosive eruptions^24^, whereas the eruption on 21 November was a small-volume phreatomagmatic event. For this reason, we favor interpretation of extensive scrubbing during shallow magma residence prior to eruption.

Following the first phreatomagmatic eruptions, SO_2_ emission rates were high on 22 November (660 t/d) but waned rapidly. Plume CO_2_/SO_2_ increased by an order of magnitude from 23-24 November (77 to 824) but this change in the ratio was due to lower plume SO_2_ abundances, rather than an increase in CO_2_. Most likely, the pathway for magma degassing was temporarily opened after the explosions on 21 November, but soon closed so that gas again traversed the extensive groundwater system with its capacity to dissolve SO_2_. The process repeated on November 25 when renewed phreatomagmatic explosions gave way to magmatic explosions and gases rich in SO_2_ were again measured. The eruption plume heights peaked on 27 November, coincident with very high lava effusion rates, reaching 36 m^3^/s over the interval 25–29 November. Peak SO_2_ emission rates were measured during this interval as well, with 5,500 t/d measured on 26 November. Lava effusion slowed dramatically by 30 November, but small explosive eruptions continued, venting through surface lava such that they were not recorded seismically. By 23 December, the character of these explosions had changed. Explosions began to produce permanent pits in the lava surface and were detectable seismically, a change that we interpret to reflect rheological stiffening of surface lava due to cooling and coincident solidification. Furthermore, large decreases in SO_2_ emission rates and increases in BrO/SO_2_ after late November were consistent with diminishing eruption rates through time (to 230 t/d average in January and 220 t/d in February), and a dominance of shallow degassing.

Using this framework, we now favor a conceptual model that includes dike emplacement and involves several stages of magma ascent:

1. Early magma ascent in February – March 2017 from a deep crustal reservoir (possibly rising from a reservoir near the crust-mantle boundary into the mid-crust (12 to >15 km; Fig. 7B; *c*.*f*. ref.^20^) followed by an additional similar intrusion in late August to mid-September 2017. Both events were evident with inflationary signals in GNSS data, but neither was detected with InSAR interferograms. The initial deep intrusion was aseismic, though the second was accompanied by some VT seismicity.
2. Magma intruded as a dike located between Mount Agung and Batur caldera, at a depth ~ 10 km^10^. This intrusion took place in late September to early October 2017, as detected both by the GNSS network and InSAR interferograms and was accompanied by high rates of VT seismicity in the area of the inferred dike (Fig. 7B). During this time, SO_2_ emissions remained low, though some increased heat and steam emerged at Mount Agung. On 20 October seismicity decreased sharply but VT events started to shallow.
3. Beginning around 12 November, magma ascended into the shallow system, as inferred based on changes in seismic event types (from VT to LF and tremor) and locations (from distal to proximal with respect to Mount Agung). We speculate that the M4.9 earthquake on 8 November marked the culmination of pressurization due to the intrusion, and that magma later was able to progress upward through a more permeable pathway toward the surface. Toward the end of the period 12–22 November, magma reached shallow depths (<1–2 km) and CO_2_ was detected with multi-GAS drone flights, but SO_2_ was not detected during DOAS traverses due to scrubbing by the groundwater system. We currently lack both seismic and geodetic data to constrain the shallow plumbing system for final ascent of magma to the surface.
4. The eruption began on 21 November with phreatomagmatic explosions, and high SO_2_ emissions were measured. On 25 November a small lava flow was detected in the crater, coincident with continued explosions. By the end of November, a 24 million m^3^ lava flow filled about one-third of the crater volume. Ash-rich explosions through the lava continued over subsequent months. Initially, cracks and explosion craters that formed in the flow were filled with lava, but as the flow stiffened with time, subsequent cracks remained unhealed. Over time, as the magma eruption rate declined, so did gas emissions.

#### Event trees to assist in eruption forecasting

To assist with forecasting and providing advice to emergency managers, we utilized probabilistic event trees eight times during the Mount Agung crisis: on 20 September, 23 September, 2 October, 17 October, 15 November, 11 December, 24 January, and 12 March (Table 2). The event trees were populated by subjective combination of relevant data to facilitate estimation of the relative probabilities of a range of outcomes, including eruption size (VEI), products (lava flow, lahar, PDC, tephra), and impacts (distance, characteristics). Event probabilities were based on the discussion and expert-elicitation process used by the VDAP and CVGHM team^25,26^. All event trees and their supporting data are presented in the Event-Tree Supplement. Each of these short-term, event-tree forecasts covered a period of two weeks, because this relatively short time interval was of relevance to the emergency managers. Early event-tree eruption forecasts (late September and early October) relied heavily on seismic data, remote sensing observations, analysis of past eruptive periods at Agung, and comparisons with analog volcanoes and analog seismic progressions. Table 2 shows the likelihood of eruption estimated as 50%, 70%, and 60%, on 20 September, 23 September, and 2 October respectively, where values correlate with seismic event rates. Uncertainties were subjectively placed at ±10% due to lack of deformation and gas data and limitations of using two short-period seismic stations (located 4–5 km from the summit) to interpret apparent lack of LF or shallow seismic activity. All three of these trees placed equal conditional probabilities of VEI 0–1 and VEI 2–3 (each at 45%) scenarios over the 2-week interval and only a 10% probability of a VEI 4 or greater (i.e., another 1963-size event), if an eruption were to occur. These probabilities were based on the global eruption magnitude-frequency distributions modified by the current rate and style of seismicity, and analogy with characteristics of the 1963 eruption.

**Table 2:** Probability-tree exercises to assist eruption forecasting.

Date	Probability of eruption in 2 weeks	Probability eruption increases (2 weeks)	Probability eruption stay same (2 weeks)	Probability eruption lessens (2 weeks)	Maximum VEI 1 (2 weeks)	Maximum VEI 2 (2 weeks)	Maximum VEI 3 (2 weeks)	Maximum VEI ≥ 4 (2 weeks)
20-Sep	0.50 ± 0.10				0.45	0.45		0.10
23-Sep	0.70 ± 0.10				0.45	0.45		0.10
02-Oct	0.60 ± 0.10				0.45	0.45		0.10
17-Oct	0.50 ± 0.20				0.45	0.25	0.20	0.10
15-Nov*	0.40 ± 0.20				0.45	0.25	0.20	0.10
*Post-Eruption Initiation*								
11-Dec		0.55	0.40	0.05		0.55	0.28	0.17
24-Jan		0.20	0.70	0.10		0.75	0.20	0.05
12-Mar		0.25	0.60	0.15		0.90	0.09	0.01

*In only the 15-Nov event tree, the relative likelihood that eruptions would be phreatic vs. magmatic was added to the tree and estimated at 80%:20%.

As the crisis continued (mid-October to November), additional monitoring streams aided short-term forecasting efforts, including the GNSS data retrieved in early to mid-October and initial InSAR data that were processed, corrected, and interpreted by mid-November. Eruption likelihoods were estimated at 50% and 40% on 17 October and 15 November, respectively, as time since peak seismicity increased and the possibility of waning activity was increasingly considered. However, uncertainty (±20%) also increased over previous trees due to slow rates of change in seismicity and increasing duration of unrest without eruption. Conditional probabilities for eruption magnitude remained the same as in earlier trees because their basis remained the same, but were subdivided, placing VEI 0–1 at 45%, VEI 2 at 25%, VEI 3 at 20%, and VEI 4–5 at 10%. The 15 November tree further highlighted a phreatic eruption scenario, which was deemed to be the most likely (80%) conditional eruptive scenario. Both trees preceded the addition of drone-assisted multi-GAS geochemical measurements, the results of which would have increased expert estimation of eruption probability, and the first of which was rapidly followed by eruptions on 21 November.

For all of these event trees, forecasts of PDC runout relied on comparisons with global data from the FlowDat database^27,28^, energy cone models, and past runout of PDC’s during the 1963 eruptive sequence. Due to the large size and steep slopes of the Agung edifice, even relatively small volume eruptions can produce PDCs with far-travelled runout. Additional information is available in the Event-Tree Supplement).

### Challenges in successful eruption forecasts

Our ability to forecast upcoming activity was initially limited by the number of operational seismic and GNSS stations, the hazard in climbing to the summit to make observations, and clouds and rain inhibiting robust DOAS measurements of the low gas output. Over time, these challenges were solved by addition of seismic and GNSS stations, the use of drone-assisted multi-GAS measurements, radar satellite surveillance, and event trees. InSAR, though highly important toward our current understanding, was a challenge to use during the crisis because of the relatively small geodetic signal, and the requirements for careful subtraction of tropospheric humidity effects on the radar signal that introduces latency between data collection and confidence in the processed data sufficient to inform hazards assessments.

Another key aspect of unrest at Mount Agung was the long two-month interval between peak seismicity and eruption, which clearly affected the results of the group forecasts through the event-tree exercise (Table 2). What additional information does this delay time provide about forecasts? Is delay time an inverse proxy for ascent rate and thereby degree of pressurization of the system, such that increasing delay implies a lesser eruption intensity? If delay between peak seismicity and magmatic eruption is a proxy for ascent rate, instead of decreasing probability of eruption with time after peak seismicity, perhaps a decrease in likely size of eruption may be implied. Furthermore, global data of delay/run-up times between seismicity increase and eruption^29^ range from minutes to months in mafic systems. We suggest that a careful evaluation of delay times compared to eruptive outcomes may help inform the most appropriate time interval over which short-term forecasts should be made.

Finally, we are still puzzled by the relative lack of shallow seismicity during final ascent compared to intrusion of the inferred dike at ~10 km depth^10^. Is it possible that this difference was due to magma ascent-rate variation, where final ascent was slower than transfer into the inferred 10-km-deep dike? Or, is it possible that the permeabilities and rheologies of the pathways are sufficiently different to account for the variation in seismic behavior?

## Crisis Response

### Social and political situation

A number of factors contributed to intense social and political pressure on those involved in the response to the 2017-2018 crisis. These factors include: (1) a history of large eruptions at Mount Agung^7^; (2) the large population within the hazard zones (~200,000); (3) the high population density of eastern Bali; (4) Bali’s importance as a domestic and international tourist and business conference destination; (5) the presence of important agricultural and aggregate resource areas on the flanks and upper slopes of Mount Agung; (6) fifty years of dormancy at Mount Agung accompanied by minor eruptions at the nearby Batur caldera; (7) a majority population rooted in the stoic and ritualistic philosophy of Balinese Hinduism; and (8) the relatively long run-up time of ten weeks between initial felt earthquakes and magmatic eruption.

### Alert-levels and exclusion zones

It has been argued that linking alert levels to mitigation actions can result in volcanologists’ decisions being too affected by social and political forces^30^, yet such a linkage has been practiced for decades in Indonesia and has saved many tens of thousands of lives^31–33^. The Agung crisis is an example of how, despite intense social and political pressure, through coordinated actions of the volcanologists at CVGHM and members of the Indonesian emergency management community, mitigation actions were taken that effectively protected the at-risk population from what could have been a major disaster.

As in many countries, Indonesia uses a type of Incident Command System^34^, in which a volcano observatory issues warnings and acts as the primary advisor to the emergency managers. In Indonesia, CVGHM is responsible for both alert levels and hazard zonation maps; whereas, the Badan Nasional Penanggulangan Bencana (BNPB, National Disaster Management Agency) along with Badan Penanggulangan Bencana Daerah (BPBD, Regional Disaster Management Agency) and local government agencies use the CVGHM alerts and maps to prepare response plans. Evacuations are organized by these emergency managers and carried out by various combinations of national and local military and police agencies and by community leaders. Implementation of mitigation policies, strategies and actions by governments and communities are directly tied to the four alert levels (Fig. 1B, Table 1). In addition, mandatory actions are required of agencies at certain alert levels and funding is allocated by national and regional governments to support such actions (e.g., for evacuation camps). CVGHM also has a mandate to facilitate and support community-level capacity building. Consequently, congruence of mitigation programs between CVGHM and local governments is required for success, and related protocols are enacted in public law^31^.

CVGHM’s 4-level alert system is based on the extent of unrest at the volcano and the potential hazards at each level. Community responses to the alert levels are developed in partnership with civil protection authorities and are linked to specific Hazard Zones that are specified on maps produced by CVGHM. This Indonesian approach to alerting and partnering with civil protection authorities has been proven effective in saving tens of thousands of lives^31–33^. A distinction between the highest two alert levels (“Siaga” or watch, and “Awas,” or warning) is whether there are indications that eruptions could potentially threaten populations near the volcano. This distinction, which clearly goes beyond the capabilities of volcanologists alone^30^, requires an effective partnership between CVGHM and the Indonesian emergency management and political community. Although the primary factor in decisions to change the alert level at Agung was the state of volcanic unrest as determined by CVGHM, many other factors related to the potential threat to population were considered by the emergency managers. There was considerable pressure on CVGHM to lower the alert level following the initial decline in seismicity in early October through the first eruption, and during the extended period December 2017–early February 2018 (Fig. 2). Factors that played a role in maintaining the alert level at Awas and Siaga included the continued activity and possibility of a larger eruption (Table 1), coupled with a number of social factors.

During the Mount Agung crisis, CVGHM issued formal written advisories and recommendations to the emergency management community and to government officials at all levels. At Alert Levels 3 and 4, these advisories were issued every 6 hours. A sector-based hazard zonation and associated evacuation system was updated and used as illustrated in Fig. 1B. The exclusion zones were largely based on the modeling of pyroclastic density currents (using energy cones and TITAN2D^35,36^, lahars (using LAHARZ^37^, and ash dispersal (using ASH3D^38^ coupled with the existing CVGHM hazard map^39^ (Fig. 1B). Ballistic impact zones were also considered using data from the 1963 Mount Agung eruption^1–4^, as well as analog volcanoes.

The modeling of PDCs from a VEI 3 scenario, the existing hazard map and the 1963 deposit distribution greatly influenced the initial large (9–12 km) exclusion zone for Level 4 implemented on 22 Sept. (Fig. 1B). The relatively large exclusion zone represented a conservative approach to the hazards, given that the most likely eruption, based on probabilistic event-tree forecasts, was for a smaller (<VEI 3) event. At some Indonesian volcanoes with more frequent and smaller eruptions, the most likely (or typical) magnitude of eruption has typically been used for mitigation strategy. However, one of the lessons learned at Merapi in 2010, was that larger, non-typical, events need to be included in mitigation plans to minimize losses^32,33,40^ even if such plans result in some false alarms^41^.

### Communications protocols and technologies

The increasing activity of Mount Agung reinforces our experience that public communication is critical during crises. CVGHM extensively used the MAGMA Indonesia application as a tool to disseminate information during the Mount Agung crisis (Supplemental Figures Figs S2 and S3). The application, which is accessible on the internet (https://magma.vsi.esdm.go.id/) and optimized for mobile smart phones, provides rapid communication of a wide range of geological hazards information from CVGHM to the public. It disseminates varied information, including volcano alert levels, observation reports with hazard warnings and mitigation recommendations, hazard maps, press releases, volcano observatory notice for aviation (VONA) alerts, and near-real-time monitoring data (e.g., seismograms and web-cam imagery). The application also allows users to share information through various social media (e.g. WhatsApp, Facebook, Twitter, Instagram, etc). Advisories for Agung were provided to the public, mainly in Indonesian, but also at critical times in English to meet demands for information to the international tourist community. The Agung volcano observers routinely used this application to disseminate volcanic activity reports to the communities and stakeholders through tens of WhatsApp (a messaging application) groups. WhatsApp was the most used messaging application for most Indonesians to share information at that time.

An innovative multiplex radio-based system was used during Alert Level 4, in which “instant warnings” could be delivered simultaneously to the dozens of community officials on the flanks of the volcano. The radio system was provided by the Indonesian Amateur Radio Organization (Orari) Using the Orari system, warnings of unrest that exceeded predefined parameters could be issued immediately, even by a single observer at the Agung Observatory in the middle of the night. The messages used a simple single-digit numbered system to advise on the extent of the hazard. For example, the simple coded message “Warning #4” indicated a hazard of PDC’s that could potentially extend to a radius of 18 km in predefined sectors of the volcano. Crisis response officials in all communities on the flanks of the volcano routinely use radios to communicate, therefore the radio system was a familiar and accepted means to receive hazard warnings. The officials receiving the messages were trained about the hazards and how to respond, and they had maps showing evacuation zones for each level of instant warning.

### Evacuations, socialization and community response

When CVGHM increased the alert level on 14 September from Normal (Level I) to Waspada (Level II), people’s awareness started to increase. While most of the people were aware of the hazard warnings, most of them were not aware of how to respond to such warnings. Most people did not have sufficient knowledge regarding hazards and risks around the volcano, especially given that the volcano had been dormant for over fifty years. This was shown during the rapid changes in alert levels from Waspada (Level II) to Siaga (Level III) on 18 September and from Siaga (Level III) to Awas (Level IV) on 22 September, resulting in a chaotic situation for the community around the volcano. Moreover, false information was circulated amongst the local (and global) population, stating that an eruption was imminent. People hurriedly evacuated and were stuck in traffic for tens of hours on their way to evacuation camps. Some felt the need to sell their livestock at a loss. Some people unnecessarily self-evacuated based on their understanding of the events of 1963, and not based on the actual exclusion zones. Although only around 70,000 people from 27 villages were supposed to be located in the evacuation camps, an additional >70,000 people from 51 villages self-evacuated from outside of the recommended exclusion zone. This created a more expensive and chaotic situation that added to the challenge for public officials and emergency response agencies.

When the eruption did not occur soon after the alert level was first elevated to the highest level (Awas), there was a growing opinion that the CVGHM volcanologists had made a mistake in analyzing the activity. After the alert level was downgraded and the exclusion zone was reduced in distance in October (6.0–7.5 km from the crater), some people came back to their homes and some believed that the volcano would not erupt. However, in one month the volcano eventually erupted, the alert level was upgraded, the exclusion zone was increased (8–10 km from the crater), and some people had to return to evacuation shelters, creating further confusion and frustration. Clearly, it is difficult to balance the level of concern in the local population with the perceived risk by the volcano observatory, and with the mitigation responsibilities of the many relevant local and national governmental agencies.

In order to relieve tensions and to increase people’s understanding related to Mount Agung’s dynamic activities over the course of the crisis, CVGHM undertook a series of strategic actions. Balinese people, especially those living around Mount Agung, are deeply influenced by their culture and religion; furthermore, community and religious leaders have a key role in public communication. CVGHM responded by organizing regular direct discussions with these leaders at local government offices or at residences of community and religious leaders. As these meetings progressed over time, mutual trust was built. We found that these leaders had a decisive role in improving communications between CVGHM and the local community. Direct discussions with the community were also carried out in refugee shelters and were attended by several related stakeholders including local government agencies, Regional Disaster Management Agency (BPBD), army, police, etc. CVGHM often utilized native Balinese spokespersons to more fully relate to the local population. CVGHM scientists also gave hundreds of interviews to the news media. Although this required a major effort in communication by scientists and officials, these actions were an essential element in maintaining public awareness of the hazards, and in explaining that the volcanic hazards did not extend to the entire island of Bali.

## Recommendations

Despite the overall success of the crisis response to the eruption of Mount Agung, there were also significant challenges. These included: maintaining the necessary resources for evacuation and community training when the alert level was not at the highest, lack of explosive eruption experience among many residents of Bali, and competing opinions from non-government officials about the relative hazards as reported in the media.

In order to maintain awareness about the hazards at Mount Agung and to mitigate the risks from future eruptions, several actions are warranted. Continual education of the local population is required to ensure that they are prepared to respond to changing events and to take recommended actions. Prior to September 2017, the local officials and religious leaders in Bali were relatively unfamiliar with the events of 1963 and the potential for renewed activity. Intensive meetings and discussions were required to allow for cooperative planning to aid in evacuation. In the future, periodic “table-top” exercises are recommended to help coordinating agencies maintain a system of communication and coordination in advance of future crises. These exercises could form a part of volcanic eruption contingency plans, which would necessitate periodic updates to reflect administrative and political changes. Although some residents remember the eruption of 1963, many others do not. Knowledge of the catastrophe was passed down through family stories, but were rarely, if ever, shared publicly. Therefore, emphasis must be placed on continuing education for local citizens and school children about volcanic hazards in the Agung area and the potential for future eruptions.

And finally, land-use planners and governing authorities should focus additional efforts into consideration of volcanic hazard zones in land management and regulatory decision-making. By limiting the population living, working, or travelling through hazard zones, the volcanic risk can be mitigated during future volcanic crises in the area.

## Supplementary information

Supplemental Figures

Cover Sheet Event-Tree Supplement

Event-Tree Supplement 1

Event-Tree Supplement 2

Event-Tree Supplement 3

Event-Tree Supplement 4

Event-Tree Supplement 5

Event-Tree Supplement 6

Event-Tree Supplement 7
