# Supplementary material for: The 2017–19 activity at Mount Agung in Bali (Indonesia): Intense unrest, monitoring, crisis response, evacuation, and eruption
Source: Sci Rep. 2019 Jun 20;9:8848. doi: 10.1038/s41598-019-45295-9 (PMC6586650; doi:10.1038/s41598-019-45295-9)
Supplement: Supplementary file 1 — Supplemental Figures [file 41598_2019_45295_MOESM1_ESM.pdf]

SUPPLEMENTARY FIGURES S1-S3

For:

The 2017–18 activity at Mount Agung in Bali (Indonesia): Intense unrest, monitoring, crisis response, evacuation, and eruption.

By: Syahbana, D.K.[1*], Kasbani, K.[1], Suantika, G.[1], Prambada, O.[1], Andreas A.S.[1], Saing, U.[1], Kunrat, S.L.[1], Andreastuti, S.[1], Martanto, M.[1], Kriswati, E.[1], Suparman, Y.[1], Humaida, H.[1], Ogburn, S.[2], Kelly, P.[2], Wellik, J.[2], Wright, H.[2], Pesicek, J.[2], Wessels, R.[2], Kern, C.[2], Lisowski, M.[2], Diefenbach, A.[2], Poland, M.[2], Beauducel, F.[1, 3, 4], Pallister, J.[2], Vaughan, R.G.[5], Lowenstern, J.B.[2]

[1] Center for Volcanology and Geologic Hazards Mitigation, Geological Agency, Ministry of Energy and Mineral Resources, Bandung, Indonesia
[2] U.S. Geological Survey, Volcano Disaster Assistance Program, Vancouver, WA, USA
[3] Institut de Physique du Globe de Paris (IPGP), Paris, France
[4] Institut des Sciences de la Terre (ISTerre/IRD), Grenoble, France
[5] U.S. Geological Survey, Astrogeology Science Center, Flagstaff, Arizona, USA

*Corresponding Author: devy.syahbana@gmail.com

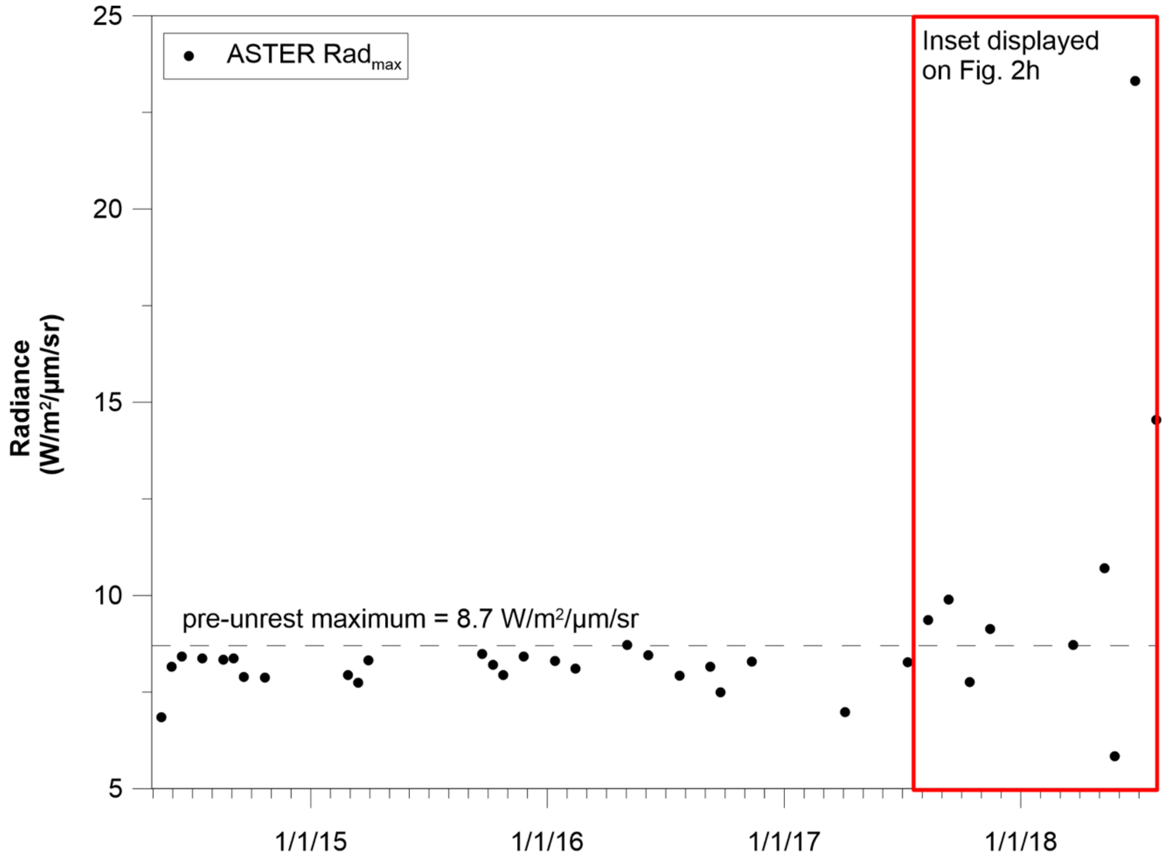

Figure S1. Complete series of ASTER maximum radiance values from the crater, with pre-unrest maximum radiance (8.7 W/m₂/μm/sr) plotted as dashed line. Red inset box shows the portion of data plotted on Figure 2h. The increase in thermal radiance beginning in August 2018 corresponded to an increase in fumarolic activity coincident with peak seismic unrest.

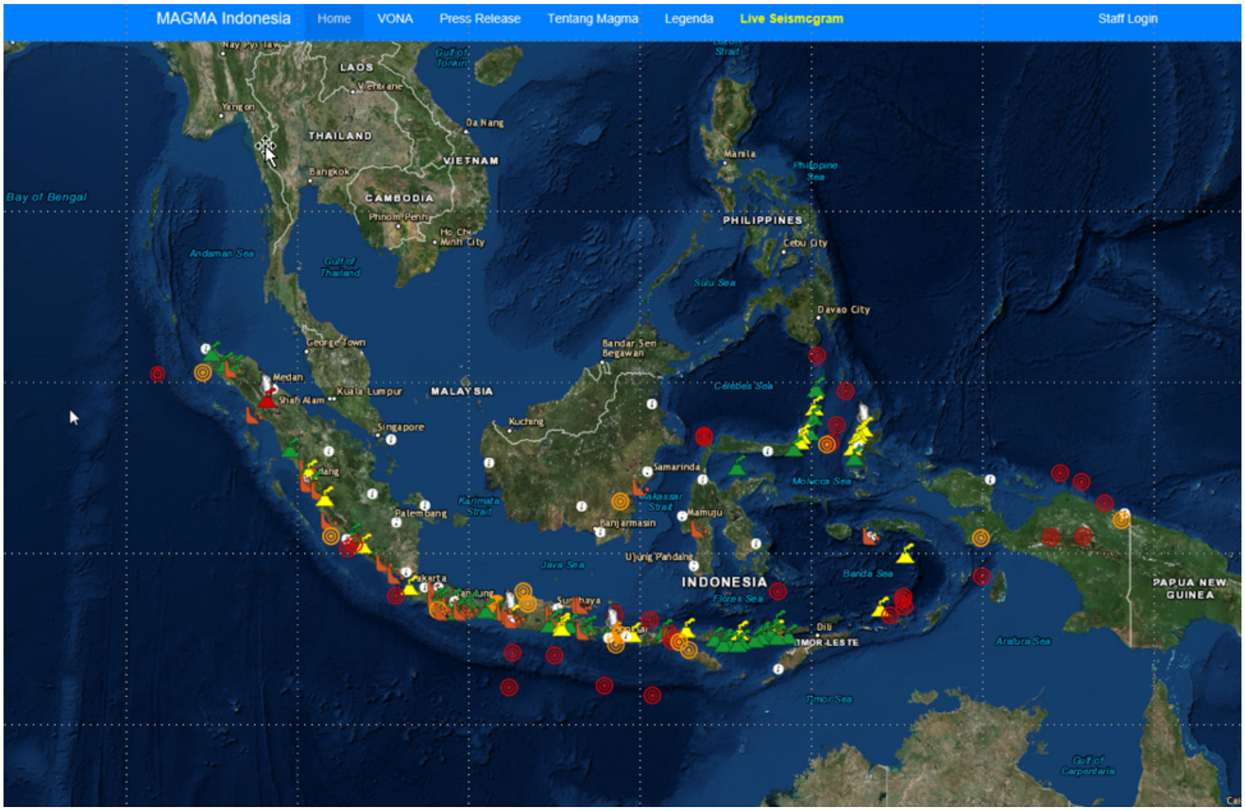

Figure S2. Home page of the MAGMA Indonesia web application, showing an index map of Indonesia region, with symbols for all volcanoes (color coded for Alert Level), as well as earthquake epicenters and landslide locations. This image shows activity on 23 May 2018.

Change in Mount Agung Alert from Level IV (WARNING) to Level III (WATCH)

12 February 2018 10:53 WIB, Ir. Kasbani, M.Sc.

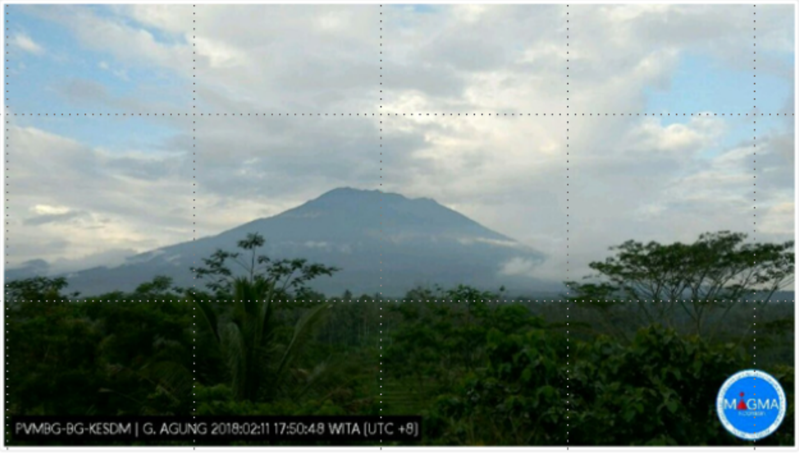

The following are our evaluation points on Mount Agung latest activities as of February 10, 2018.

A. Multi-Parameter Data Analysis

1. The eruption frequency has been in decline. The last eruption was on January 24, 2018, which was 17 days ago. The ash column reached 1000 meter above the summit. In the past 30 days, the highest eruption column was approximately 2500 meter above the summit. This took place 22 days ago on January 19, 2018.
2. The volume of the lava dome on the surface of the crater has not changed significantly, remaining at approximately 20 million cubic meters.
3. The number of volcano-related earthquakes has been lessening. Volcanic earthquakes had escalated occasionally, but not significant enough. This indicated that the magma movement is still taking place around inside the volcano but with low intensity.
4. GPS measurements exhibited relatively stable patterns while tiltmeter measurements showed slight inflationary patterns. Taken together, they pointed to plausible pressure accumulation, but without significant enough intensity.
5. Measurements on volcanic gas emissions on the volcanic plume indicated that magmatic activities had been ongoing but with lower intensity in comparison to the corresponding values measured around the time of the eruption in end of November 2017.
6. Thermal satellite images showed a decrease in the surface temperature of the lava deposit on the crater. This suggested that the flow of lava towards the surface may had decreased, which might point to either there were clogings on the upper portions of the magma pipe or that the actual flow of the magma towards the surface had declined.

B. Potential Hazards

1. At current conditions, primary hazards may potentially include ballistic projectiles formed by pyroclastic material as well as dense ash fall around the crater. Lower intensity of volcanic ash may spread fast and wide, depending on the wind direction and velocity.
2. Secondary hazards may occur in the form of rain-triggered lahars especialy during the rainy season as long as volcanic materials from previous eruptions remain in the vicinity of the summit area. Areas with heightened risk of rapid lahars include rivers/waterways whose headwaters are sourced in Mount Agung, especially those flowing to the North, North East, South East, South, and South West.

C. Conclusion

1. The data indicated that the volcanic activities of Mount Agung had decreased but not died down completely. There remains some potential for eruptions, but with lower explosivity index. Thus, we strongly advise all stakeholders concerned to maintain watchfulness as volcanic activity is very dynamic and may change at any time.
2. Based on the data analysis and the recent assessment of Mount Agung potential hazards, **at 09:00 local time on February 10, 2018, we lower the alert on Mount Agung from Level IV (WARNING) to Level III (WATCH).**

D. Points of Recommendations

1. Residents living around Mount Agung, climbers, visitors, and tourists should not stay or perform any kind of activities in the hazard zone that includes the crater and the area within a 4 km radius from the summit crater. The area defined as the hazard zone is dynamic and may change at any time depending on the latest observations.
2. Those who reside and or conduct activities on or near the rivers/waterways with headwaters around Mount Agung should beware of hazards posed by lahars, especially when it rains.
3. Considering the hazards posed by volcanic ash on human respiratory system, residents living around the volcano should have masks that provide sufficient protection on the nose, mouth, and eyes.
4. Local government agencies, the National Agency for Disaster Management (BNPB), and other relevant agencies must maintain excellent communication lines to mitigate the effects of any potential eruption by disseminating accurate information in timely manner.
5. All stakeholders in the aviation sector must routinely monitor the activities of Mount Agung due to the possibility of rapid changes in observational data that potentially affect the safety of flights.
6. All must refrain from spreading any hoax, false, or unverified information from dubious sources on Mount Agung vocanic activities.
7. The Center for Volcanology and Geological Hazard Mitigation in coordination with the National and Local (Provincial and Regency-Level) Agencies for Disaster Management is in charge of disseminating reliable information regarding Mount Agung activities.
8. Residents of areas surrounding the volcano, climbers, visitors, and tourists are to remain calm and watchful. They must follow the directives issued by the local government agencies upon the recommendation of the Center for Volcanology and Geological Hazard Mitigation to ensure that strategic mitigation efforts are performed as soon as needed.
9. Everyone can monitor the current status of and recommendations on Mount AgunG activities via the MAGMA Indonesia webpage https://magma.vsi.esdm.go.id or by downloading the Android App MAGMA Indonesia from Google Play Store. You can actively report incidents connected to volcanic activities by using the Lapor Bencanaâ€™ feature. Aviation stakeholders may access the VONA (Volcano Observatory Notice for Aviation) feature.

Source: Center for Volcanology and Geological Hazard Mitigation, Geological Agency, Ministry of Energy and Mineral Resources, Republic of Indonesia

Figure S3. Example of Alert Level Change from Level IV to Level III within the Magma Indonesia website for the change made on 10 February 2018.
