# Supplementary material for: The 2017–19 activity at Mount Agung in Bali (Indonesia): Intense unrest, monitoring, crisis response, evacuation, and eruption
Source: Sci Rep. 2019 Jun 20;9:8848. doi: 10.1038/s41598-019-45295-9 (PMC6586650; doi:10.1038/s41598-019-45295-9)
Supplement: Supplementary file 2 — Cover Sheet Event-Tree Supplement [file 41598_2019_45295_MOESM2_ESM.docx]

EVENT TREE SUPPLEMENT

**The 2017–18 activity at Mount Agung in Bali (Indonesia): Intense unrest, monitoring, crisis response, evacuation, and eruption.**

By:  Syahbana, D.K.^1*^, Kasbani, K.^1^, Suantika, G.^1^, Prambada, O.^1^, Andreas A.S.^1^, Saing, U.^1^, Kunrat, S.L.^1^, Andreastuti, S.^1^, Martanto, M.^1^, Kriswati, E.^1^, Suparman, Y.^1^, Humaida, H.^1^, Ogburn, S.^2^, Kelly, P. ^2^, Wellik, J. ^2^, Wright, H. ^2^, Pesicek, J. ^2^, Wessels, R.^2^, Kern, C. ^2^, Lisowski, M. ^2^, Diefenbach, A.^2^, Poland, M.^2^, Beauducel, F.^1, 3, 4^, Pallister, J.^2^, Vaughan, R.G.^5^, Lowenstern, J.B.^2^

^1^ Center for Volcanology and Geologic Hazards Mitigation, Geological Agency, Ministry of Energy and Mineral Resources, Bandung, Indonesia

^2^ U.S. Geological Survey, Volcano Disaster Assistance Program, Vancouver, WA, USA

^3^ Institut de Physique du Globe de Paris (IPGP), Paris, France

^4^ Institut des Sciences de la Terre (ISTerre/IRD), Grenoble, France

^5^ U.S. Geological Survey, Astrogeology Science Center, Flagstaff, Arizona, USA

*Correspondence to [devy.syahbana@gmail.com](mailto:devy.syahbana@gmail.com)

**This series of Excel Workbooks represents the event-tree exercises undertaken to assist with eruption forecasting at Mount Agung in 2017 and 2018. The notes within the workbooks are unedited and are only provided to give an understanding of the process used by scientists to assist with documentation and forecasting.**
